# Genome-wide mapping and profiling of γH2AX binding hotspots in response to different replication stress inducers

**DOI:** 10.1186/s12864-019-5934-4

**Published:** 2019-07-12

**Authors:** Xinxing Lyu, Megan Chastain, Weihang Chai

**Affiliations:** Department of Biomedical Sciences, Elson S. Floyd College of Medicine, Washington State University, Spokane, Washington USA

**Keywords:** γH2AX, Replication stress, Genome stability, Fragile sites

## Abstract

**Background:**

Replication stress (RS) gives rise to DNA damage that threatens genome stability. RS can originate from different sources that stall replication by diverse mechanisms. However, the mechanism underlying how different types of RS contribute to genome instability is unclear, in part due to the poor understanding of the distribution and characteristics of damage sites induced by different RS mechanisms.

**Results:**

We use ChIP-seq to map γH2AX binding sites genome-wide caused by aphidicolin (APH), hydroxyurea (HU), and methyl methanesulfonate (MMS) treatments in human lymphocyte cells. Mapping of γH2AX ChIP-seq reveals that APH, HU, and MMS treatments induce non-random γH2AX chromatin binding at discrete regions, suggesting that there are γH2AX binding hotspots in the genome. Characterization of the distribution and sequence/epigenetic features of γH2AX binding sites reveals that the three treatments induce γH2AX binding at largely non-overlapping regions, suggesting that RS may cause damage at specific genomic loci in a manner dependent on the fork stalling mechanism. Nonetheless, γH2AX binding sites induced by the three treatments share common features including compact chromatin, coinciding with larger-than-average genes, and depletion of CpG islands and transcription start sites. Moreover, we observe significant enrichment of SINEs in γH2AX sites in all treatments, indicating that SINEs may be a common barrier for replication polymerases.

**Conclusions:**

Our results identify the location and common features of genome instability hotspots induced by different types of RS, and help in deciphering the mechanisms underlying RS-induced genetic diseases and carcinogenesis.

**Electronic supplementary material:**

The online version of this article (10.1186/s12864-019-5934-4) contains supplementary material, which is available to authorized users.

## Background

Faithful and complete DNA replication is vital for cell survival and genetic transmission. Replication fork progression is constantly challenged and may be stalled by environmental insults and endogenous stress arising from normal cellular metabolism, leading to replication stress (RS) [1–3]. These challenges can arise from various genotoxic mechanisms, such as depletion of nucleotide pools, deficiency of replication complex, conflicts between replication and transcription, R-loop formation, DNA damage, and others (reviewed in [3]). Replisomes need to overcome these obstacles in order to complete DNA replication in a timely and accurate manner.

Fork stalling elicits the activation of the ATM- and Rad3-related (ATR) kinase, a member of the phosphoinositide 3-kinase (PI3K)-like protein kinase [4]. ATR activation arrests cell cycle, promotes fork stability to prevent fork collapse, and regulates DNA repair pathways to rescue stalled forks. One of the critical downstream target of ATR is histone H2AX [5]. Phosphorylation of H2AX at the serine residue 139 (γH2AX) by ATR is an early event in response to fork stalling [6]. Once phosphorylated, γH2AX marks stalled forks prior to DSB formation [6], presumably setting up a favorable chromatic environment that facilitates the recruitment of fork repair proteins to stalled sites. γH2AX also accumulates at break sites after fork collapse [6–8], consistent with its function in double-strand break (DSB) repair. The importance of γH2AX in fork rescue is supported by the yeast study demonstrating that a mutant of the *HTA* gene that abrogates γH2A (γH2AX ortholog in yeast) confers hypersensitivity to camptothecin, a potent inhibitor of the topoisomerase I that causes the collisions between topoisomerase-DNA complex and replication forks and therefore stalls replication [9]. The same mutant only shows mild sensitivivity to ionizing radiation, suggesting that γH2AX is particularly important in rescuing stalled replication.

Fragile sites (FSs) refer to chromosomal loci that are prone to breakage upon RS. They are hotspots for genome instabilities including sister chromatid exchanges, deletions, translocations, and intra-chromosomal gene amplifications [10–15], and their instability is frequently involved in early stages of tumorigenesis [16, 17]. Due to the importance of FSs in genome stability and carcinogenesis, several methods have been developed to analyze the genome-wide distribution and characteristics of FSs. While early studies used conventional cytogenetic method (G-banding) to map FSs to regions that span megabases in human chromosomes [14, 17, 18], employment of recent sequencing technologies has allowed for fine mapping of FSs sensitive to aphidicolin (APH), hydroxyurea (HU), or ATR inhibition in various human cell lines and murine B lymphocytes [7, 19–21]. An approach using direct in situ break labeling, enrichment on streptavidin and next-generation sequencing (BLESS) has identified > 2000 APH-sensitive regions (ASRs) in HeLa cells and revealed that ASRs contain significant enrichment in satellites of alpha-type repeats in pericentromeric and centromeric regions, as well as in the large transcribed gene regions [19]. Another distinct group of FSs known as early replication fragile sites (ERFSs) have been identified in murine B lymphocytes using RPA and γH2AX ChIP-seq. ERFSs are induced predominantly in early replicating and actively transcribed gene clusters. ERFSs contain high densities of replication origins, have high GC content and open chromatin configuration, and are also gene rich [7, 22]. Nucleotide-resolution analysis of chromosome damage sites has been established with end-seq and found that long (> 20 bp) poly (dA:dT) tracts are prone to HU-induced fork collapse in mouse splenic B cells [21]. Finally, RPA ChIP-seq has identified over 500 high-resolution ATR-dependent fork collapse sites in mouse embryonic fibroblast cells, which are enriched in microsatellite repeats, hairpin-forming inverted retrotransposable elements and quasi-palindromic AT-rich minisatellite repeats, suggesting that structure-forming repeats are also DNA sequence prone to produce fork collapse [20]. However, it is worth noting that FS breakage displays cell and tissue type-specificity [23, 24], and thus it is difficult to directly compare FS location and features measured in data derived from various cell types from different organisms.

In this study, we hypothesized that different fork stalling mechanisms may stall forks at different loci and induce or exacerbate fragilities at different sequences in the genome. This, in turn, would affect the regulation and expression of different sets of genes residing within/near the fragile loci in a manner dependent on the fork stalling mechanism. For instance, fork stalling can be induced by collision between replication and transcription in large genes, R-loop formation or other replication stressors. Due to the cell type and tissue specificity of FS breakage [23, 24], this hypothesis needs to be tested in a cell type-specific manner. Here, we used ChIP-seq to map and characterize γH2AX binding sites induced by three distinct fork stalling mechanisms in one human lymphocyte cell line. The lymphocyte cell line was chosen because historically FSs have been primarily studied in cultured lymphocytes and lymphoblastoid cells. Although γH2AX spreads to large regions and its binding sites may not reflect the exact location of broken sites, mapping and characterizing γH2AX binding may still reveal important information on fragile genomic loci. Three commonly used fork stalling agents were used, namely APH, HU, and methyl methanesulfonate (MMS). APH is a DNA polymerase α inhibitor, HU is the ribonucleotide reductase inhitor that depletes nucleotide pool, and MMS is thought to stall fork progression by binding to and methylating DNA. Our γH2AX ChIP-seq mapping reveals that APH, HU, and MMS treatments induce non-random γH2AX chromatin binding at discrete regions, suggesting that there are γH2AX binding hotspots in the genome. The three treatments induce γH2AX binding at largely non-overlapping regions, supporting that different fork stalling mechanisms likely cause fork stalling at different genomic loci. We also find that γH2AX binding hotspots are depleted from CpG islands (CGIs) and transcription start sites (TSSs), but are enriched at compact chromatin regions. In addition, significant enrichment of SINEs is found in γH2AX sites in all treatments, indicating that SINEs may be a common barrier for replication polymerases. Our results provide novel insights into γH2AX binding specificity in the human genome in response to different DNA replication stressors, which will help in deciphering the mechanisms underlying carcinogenesis and RS-induced genetic diseases.

## Results

### Mapping of γH2AX binding sites induced by APH, HU, MMS with ChIP-seq

**Fig. 1:**
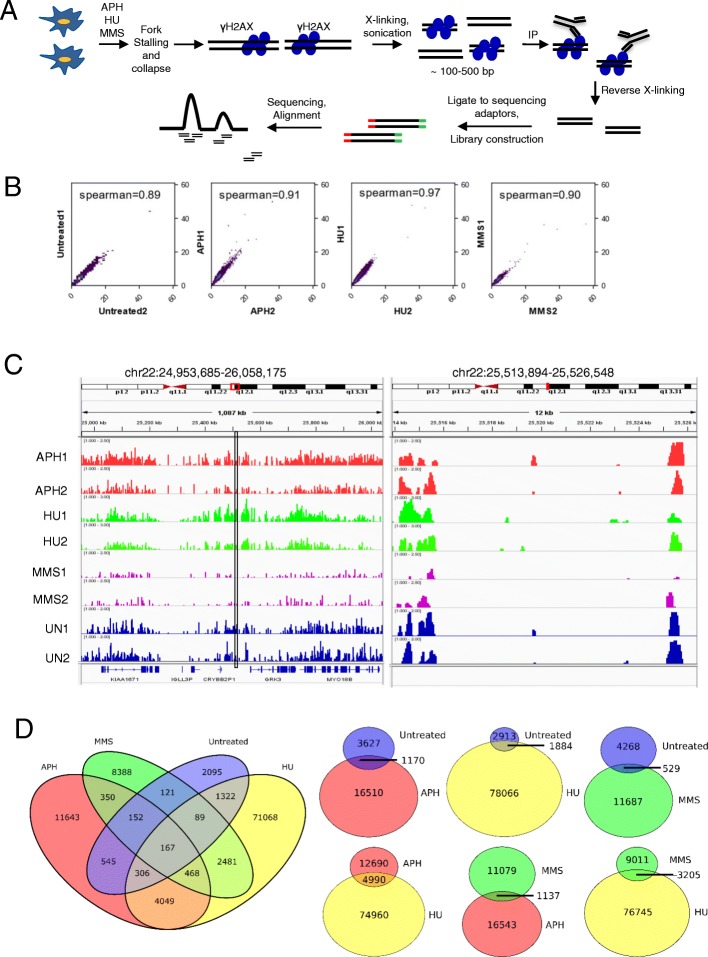
Identification of damage sites caused by fork stalling reagents using γH2AX ChIP-seq. **a** Diagram illustrating ChIP-seq experimental design. Cells were grown in suspension and treated with indicated fork stalling agents (0.3 μM APH, 2 mM HU, or 200 μM MMS) for 24 h, followed by crosslinking and γH2AX ChIP. ChIP DNA was used for Illumina sequencing. **b** ChIP-seq replicates were internally Spearman Rank Correlation between ChIP-seq replicates. Bin size 1000 bp. **c** Genome browser tracks of ChIP-seq peaks under untreated and treated conditions. Approximately 1 Mb region is shown on chromosome 22 (left) and then a 12 kb region marked with the black box is amplified (right). ChIP-seq peaks are presented after normalizing to input. Numbers in parentheses indicate fold changes in γH2AX binding relative to input. Red boxes marked on the chromosome diagrams indicate approximate genomic positions of displayed histograms. **d** Venn Diagrams depicting overlaps of ChIP-seq peaks between untreated and treated samples. Overlaps between two samples are also illustrated. While overlap does exist between samples, a large portion of all data sets are unique

Prior to ChIP-seq, we tested the specificity of γH2AX antibody to ensure high specificity of ChIP (Additional file 1: Figure S1). Exponentially growing cells were treated with APH (0.3 μM), HU (2 mM), and MMS (200 μM) for 24 h to induce RS using conditions widely reported in literatures [25–30]. Following treatment, cells were crosslinked, lysed, and DNA was sonicated to 100–500 bp. Immunoprecipitation was then performed to pull down γH2AX-bound DNA, and ChIP DNA was used for library construction and Illumina sequencing (Fig. 1a). To ensure reproducibility, two independent biological replicates were carried out, and peak calling and alignment were performed for each replicate. Since it is known that γH2AX binding to DNA spreads into large regions, broad peaks were called using MACS2 broad peak calling program [31]. Signals from ChIP samples were normalized to pre-ChIP input signals, and ChIP-seq peaks with *p* values of < 10^− 3^ were selected for further analysis. Spearman correlation coefficient between untreated and treated samples were conducted. The coefficient between replicates in each treatment was ≥0.9 (Fig. 1b and Additional file 1: Figure S2), suggesting the high reproducibility of γH2AX binding and a high confidence of ChIP-seq data. Snapshots of ChIP-seq peaks in each treatment are shown in Fig. 1c and Additional file 1: Figure S3. We observed that ChIP-seq peaks in both untreated and treated samples showed a nonrandom distribution pattern (Fig. 1c and Additional file 1: Figure S3), suggesting that these γH2AX binding sites may represent genome instability hotspots sensitive to RS.

### γH2AX binding sites induced by different stressors share little overlap

About 4700 γH2AX binding sites were identified in the untreated sample, indicating a high level of spontaneous DNA damage in this cell line. Compared to other cell lines, GM07027 displayed a high level of endogenous γH2AX expression (Additional file 1: Figure S4A). We identified ~ 18,000, ~ 80,000, ~ 12,000 γH2AX binding sites in APH, HU, MMS treated samples, respectively (Fig. 1d). We observed little overlap between APH (6.4%) and MMS (9.3%) data sets. HU treated sample contained regions shared with all other stressors, but this overlap only accounted for a small portion of the HU data set due to the number of peaks (6.2% of overlap with APH treatment and 4% of overlap with MMS treatment) (Fig. 1d).

Because HU induced four to seven times as many significant peaks as other treatments, we then checked whether such high peak number was due to the high level of damage induced by HU. All treatments induced CHK1 phosphorylation at S317, indicating ATR was activated in response to fork stalling (Additional file 1: Figure S4C). Interestingly, MMS induced strong γH2AX and CHK1 phosphorylation comparable to HU treatment (Additional file 1: Figure S4C) but showed the fewest γH2AX peaks among all three treatments. In contrast, APH induced the lowest level of γH2AX and CHK1 phosphorylation but had more γH2AX binding sites than MMS (Additional file 1: Figure S4C). These results suggest that the heterogeneity of ChIP-seq peaks produced from the three drug treatments was unlikely caused by dose effect of the stressors.

Since the drug treatments could induce cell death, and dead cells in suspension culture were difficult to remove prior to crosslinking, it was possible that damaged DNA in apoptotic cells might have given rise to γH2AX ChIP-seq peaks measured here. However, two lines of evidence strongly argue against the significant contribution of DNA damage from apoptotic cells to γH2AX ChIP-seq peaks measured here. First, if the γH2AX ChIP signals from the drug treatments were mainly from apoptotic cells, a random distribution of ChIP-seq peaks in the genome would be expected, because there are no preferred breakage sites when genomic DNA is degraded upon apoptosis. Moreover, a great overlap in ChIP-seq peaks would be expected in all three treatments. However, none of the treatments showed random γH2AX binding, and there is little overlap of γH2AX ChIP-seq peaks among three treatments (Fig. 1c, d). In addition, after performing annexin V staining to detect apoptotic cells, we found that although HU increased cell apoptosis, MMS induced comparable level of apoptosis (Additional file 1: Figure S4E). If γH2AX ChIP signals were mainly from dead cells, it would be predicted that similar numbers of γH2AX peaks in HU and MMS samples would be obtained. In striking contrast to this prediction, MMS treatment produced < 1/7 γH2AX peaks of the HU treated sample. Taken together, it is unlikely that γH2AX ChIP signals detected here were mainly from apoptotic cells.

To further understand the nature of the large discrepancy in ChIP-seq peak numbers among the three RS inducers, we then performed cell cycle analysis and BrdU incorporation assay to detect the impact of drug treatments on replication. Treated and untreated cells were pulse labeled with BrdU for 30 min prior to collection, followed by flow cytometry analysis as described in Materials and Methods. In MMS treated cells, both the number of replicating cells and BrdU intensity were similar to the untreated sample (Additional file 1: Figure S4F), suggesting a lower level of replication stress, which corresponded to the low number of observed γH2AX ChIP-seq peaks. In contrast, HU treatment dramatically hindered BrdU incorporation (Additional file 1: Figure S4F). This was expected because at the end of the 24 h HU treatment, the dNTP pool was expected to be largely depleted by HU and therefore BrdU incorporation into DNA should be minimal due to the lack of DNA synthesis substrates. This result suggests that HU treatment perhaps stalled the majority of replication forks, and thus explaining the highest γH2AX ChIP peaks in the HU sample. While APH enriched the number of replicating cells (BrdU+ cells), the majority of BrdU+ cells showed lower BrdU intensity than untreated cells, indicative of a slowdown in replication fork movement (Additional file 1: Figure S4F). This was consistent with the higher number of γH2AX peaks in APH treated sample than MMS, despite the lower level of damage and CHK1 phosphorylation (Additional file 1: Figure S4C). It is also possible that APH only stalled a subset of forks under the condition used in this study (0.3 μM). Increasing APH concentration severely interfered with the cell cycle progression and arrested the cell cycle at the late G1/early S boundary (Additional file 1: Figure S5), and therefore were not used to study RS. Together, our results suggest that the number of γH2AX ChIP-seq peaks is largely consistent with the level of RS caused by these stressors. Furthermore, the little overlap of γH2AX binding sites between each treatment suggests that γH2AX binds at specific genomic regions in a manner likely dependent on the fork stalling mechanisms.

### γH2AX binding is enriched in large genes and regions encoding long transcripts

**Fig. 2:**
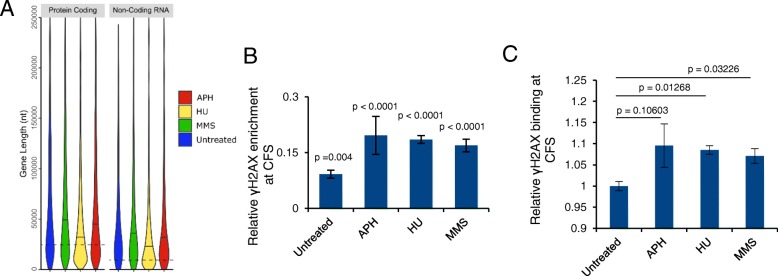
Enrichment of γH2AX in large genes and CFSs. **a** Violin plot showing γH2AX enrichment in both coding and non-coding long genes irrespective of DNA damage. Dotted lines indicate genomic median gene lengths. Solid lines indicate median gene lengths from each ChIP-seq sample. **b** γH2AX binding to CFSs is significantly higher than expected by random in both the absence and presence of exogenous DNA damaging agents. Expected γH2AX binding to CFSs by random is set to zero. Positive deviation from zero indicates enrichment. *p*-values are derived from permutation analysis. **c** HU and MMS treatment significantly increase γH2AX binding to CFSs compared to untreated cells. *p* values: Student’s t-test with Holm-Bonferroni correction for family-wise error

Our results showed that γH2AX binding was enriched at genes longer than the genomic median, regardless of the stressor (Fig. 2a and Additional file 1: Figure S6, Kruskal Wallis with post hoc paired Wilcoxan signed rank test, *p* < 2 × 10^− 16^). This result supports that large genes/transcripts have the potential to stall replication under RS induced by different treatments, presumably because replication machinery more likely collides with RNA polymerases transcribing long genes [10]. Interestingly, while HU induced γH2AX enrichment at genes longer than the genomic average, such enrichment was found at shorter genes when compared to APH or MMS treated samples (Fig. 2a, Additional file 1: Figure S6), indicating that HU treatment may sensitize shorter genes to breakage.

### γH2AX is enriched at CFSs under exogenous genotoxin treatment

Common fragile sites (CFSs) are specific chromosomal regions that are prone to break under APH-induced RS. They are present in all individuals, are characterized by gene poor, heterochromatic, late replicating, non-B-form DNA structures like hairpins [15, 32–35]. CFSs are not precisely mapped breaks, but rather are megabase regions defined by G-banding using APH treated lymphocyte metaphase spreads [14]. Using permutation analysis, we compared γH2AX enrichment at consensus CFS G-band positions (Additional file 2: Table S1). We found that CFSs accumulated γH2AX at a low level in the absence of RS and breakage was further enhanced with exogenous genotoxic stress (Fig. 2b and c). While CFSs were originally described under APH treated conditions, we found that both HU and MMS could induce significant γH2AX enrichment when compared with untreated samples (Fig. 2c). This result confirms previous findings that RS may preferentially cause damage at regions containing CFSs, and that these regions may be sensitive to a wide variety of stressors.

### Sequence features in γH2AX binding regions

**Fig. 3:**
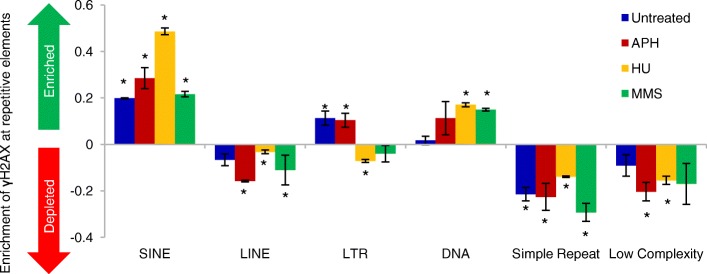
γH2AX binding to RepeatMasker defined repetitive DNA elements. Expected random γH2AX binding to a given feature is set to zero. Deviation from zero indicates enrichment (positive value) or depletion (negative value). γH2AX binding to SINEs is significantly higher than expected by random, and binding to simple repeats and low complexity repeats is lower than expected by random. * indicates *p* < 0.001 (permutation analysis)

It is thought that repetitive sequences are intrinsic barriers of replication machinery and replication forks are prone to stall at repetitive regions [36]. Thus, we analyzed ChIP-seq peaks in the context of repetitive genomic elements using the RepeatMasker data set [37]. In addition to areas of low complexity (defined as > 100 nt stretch of > 87% AT or 89% GC, and > 30 nt stretch with > 29 nt poly(N)_n_, N denotes any nucleotide, and those containing short tandem repeats [37]), we also looked at γH2AX accumulation in the context of common transposable elements: SINEs (short interspersed nuclear elements), LINEs (long interspersed nuclear elements), DNA transposons, and LTRs (long terminal repeats). No enrichment at regions of low complexity was observed. Instead, we observed significant enrichment at SINEs genome-wide in all samples (Fig. 3). SINEs are 80–500 bp nonautonomous elements in the genome, with 3′ ends often composed of simple repeats like poly-dA, poly-dT, or tandem array of 2–3 bp unit [38]. A recent study identifies that poly (dA:dT) tracts are natural replication barriers and are a common cause for DNA breakage in HU-treated mouse B-lymphocytes [21], and SINEs are significantly enriched in early replicating fragile sites identified in HU-treated mouse B-lymphocytes [7]. Another study shows that repetitive DNA sequences that give rise to non-B-form structures impede DNA replication [20]. The enrichment of SINEs but not simple repeats in γH2AX binding indicate that in addition to the 3′ poly (dA:dT), abundant transposable elements in SINEs may contain features prone to non-B-form structure formation that make SINEs particularly susceptible to fork stalling.

Compared to untreated sample, SINEs, LINEs, simple repeats, and DNA transposons were enriched in γH2AX binding sites under HU treatment, while LTRs and simple repeats were reduced in MMS treatment (Fig. 3). Binding patterns in APH treated sample did not significantly differ from untreated cells in any repetitive elements (Additional file 1: Figure S7). Future studies using a high-resolution sequencing method will be helpful to pinpoint sequence composition and features under different replication stress inducers.

### Epigenetic features in γH2AX binding regions

**Fig. 4:**
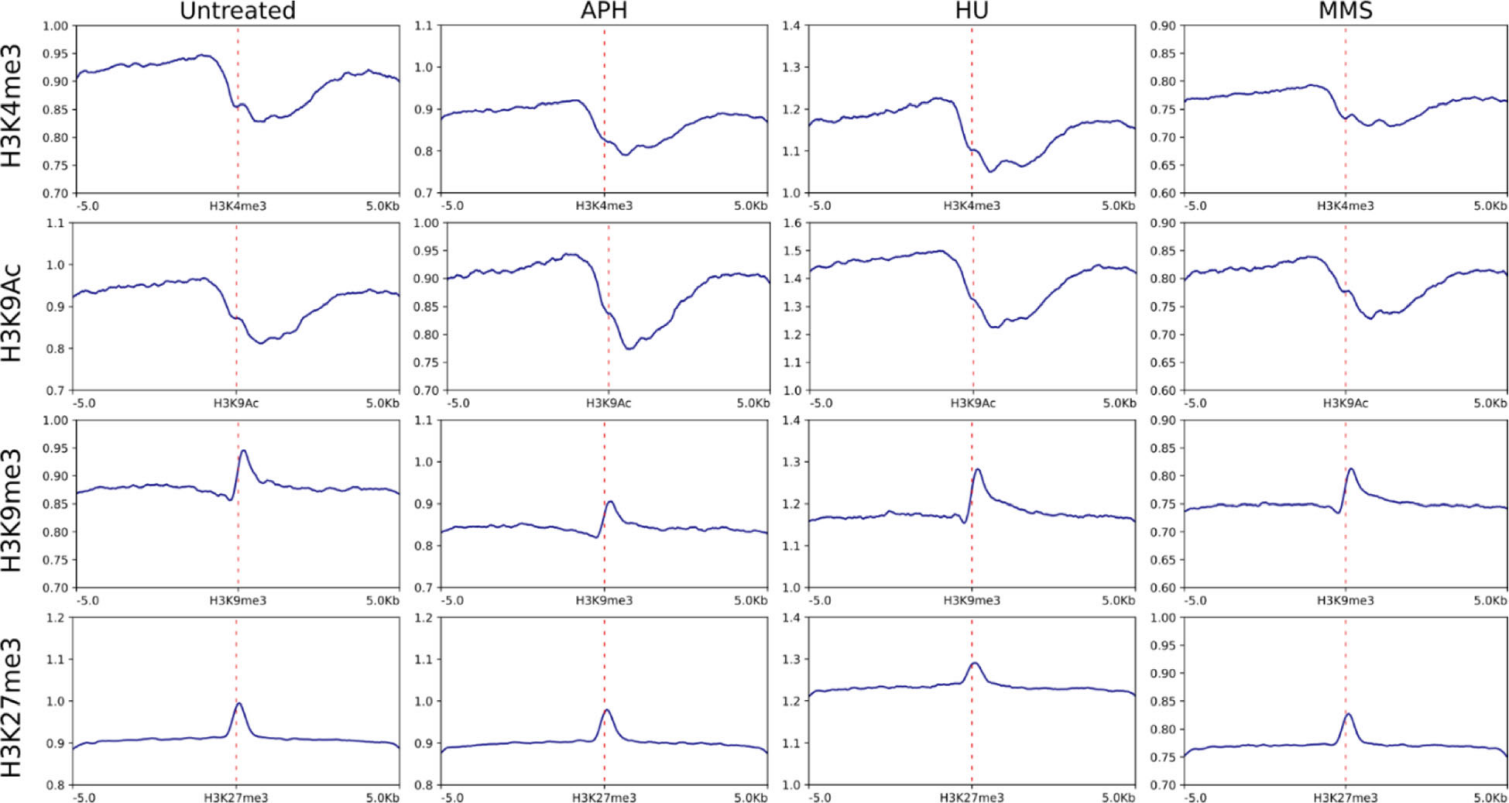
Epigenetic features in γH2AX binding regions. Average γH2AX binding relative to input was compared to the binding of indicated histone marks using published histone ChIP-seq data obtained from immortalized human B lymphocytes available in NCBI Gene Expression Omnibus. The dotted line indicates the center of the modified histone proteins, H3K27me3, H3K9me3, H3K9Ac and H3K4me3. X axis stands for these modified histone proteins distribution on the chromatin, and y-axis stands for the γH2AX binding signals corresponding to the four modified histone proteins position. γH2AX is enriched at H3K27me3 or H3K9me3 bound chromatin while depleted at H3K9Ac or H3K4me3 bound chromatin. Red dotted line indicates the center of the histone variant binding site

Poor replication initiation has been proposed to cause instabilities [35]. Given that replication timing and initiation can be epigenetically controlled rather than directed by specific sequence motif [12, 39], we examined common epigenetic marks including H3K9Ac, H3K4me3, H3K27me3, and H3K9me3 that modulate chromatin structures at γH2AX binding sites. H3K9Ac and H3K4me3 are euchromatic marks and are tightly associated with active transcription and histone deposition, while H3K27me3 and H3K9me3 are found mainly at inactive gene promoters and are associated with compact chromatin [40]. After aligning γH2AX ChIP-seq peaks with histone modification ChIP-seq datasets from human B-lymphoblastoids [GSM733677 (H3K9ac), GSM733708 (H3K4me3), GSM945196 (H3K27me3), GSM733664 (H3K9me3)], we found depletion in γH2AX at H3K9Ac and H3K4me3 marks, and enrichment in all samples at H3K27me3 and H3K9me3 marks (Fig. 4), suggesting that γH2AX sites induced by the three stressors coincide with more compact chromatin regions.

### Depletion of CGIs and TSSs in γH2AX binding regions

**Fig. 5:**
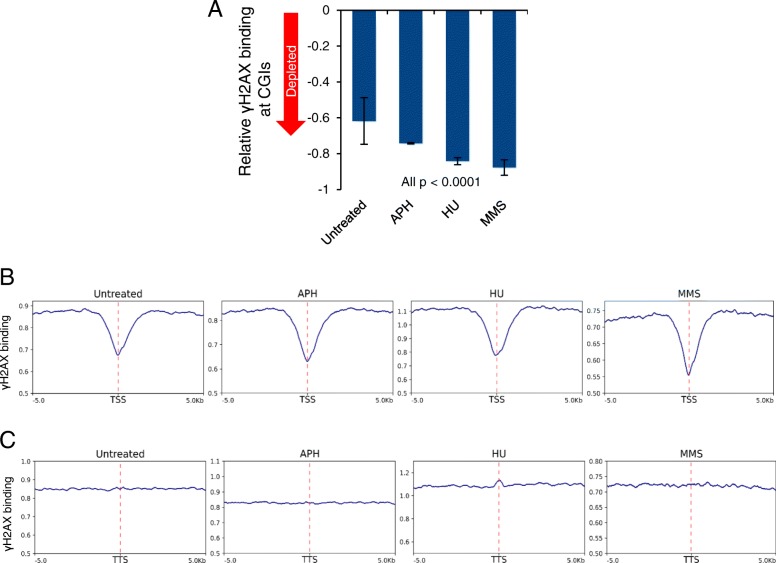
Depletion of γH2AX at CGIs, TSSs and TTSs. **a** γH2AX binding at CGIs is significantly lower than expected by random, irrespective of fork stalling agents. Expected random γH2AX binding to CGIs is set to zero. p-values: permutation analysis. **b** Average genome-wide γH2AX binding at TSSs genome-wide to input shows local depletion compared to the surrounding 10 kb region. Red dotted line indicates the TSS position. **c** Average genome-wide γH2AX binding at TTSs to input compared to the surrounding 10 kb region. Red dotted line indicates the TTS position

CGIs are DNA elements with high CpG content. Roughly 50% of these regions are associated with gene expression regulation, and can be located at or near TSSs [41–43]. Early studies have shown a strong association of replication initiation and CGIs in mammalian genomes, with half of origins residing within or near CGIs [44, 45]. Replication origin activity is also significantly enriched at and around TSSs [46, 47]. Thus, we next examined the relationship between γH2AX binding and CGIs and TSSs in our samples. Using permutation analysis, we searched for enriched or depleted binding at CGIs genome-wide and found that γH2AX did not associate with CGIs. Rather, these regions were noticeably unbound (Fig. 5a). Similarly, we found consistent local depletion of γH2AX at TSSs (Fig. 5b), while no depletion or enrichment at transcription termination sites (TTS) or gene bodies was observed (Fig. 5c and Additional file 1: Figure S8). Together with the enrichment of γH2AX binding at more compact chromatic regions (Fig. 4), our data suggest that γH2AX tends to bind to transcriptionally inactive regions upon fork stalling.

## Discussion

While γH2AX binding to DSBs has been mapped and profiled in high-resolution [48], systematic characterization and comparison of γH2AX chromatin binding in response to RS is lacking. This is further complicated by the fact that fork stalling can be induced by a diverse of mechanisms, and FS instability also displays cell- and tissue-type specificity. In this study, we generated a large set of γH2AX binding data from a single human cell line treated with three genotoxins that stall replication with distinct mechanisms. This study design allows us to directly compare γH2AX binding under different RS conditions, revealing a number of notable features of γH2AX binding in response to fork stalling.

We find that only a small portion of γH2AX binding sites resulting from MMS (9.3%) and APH (6.4%) treatment overlap, suggesting that the two different fork stalling mechanisms produce RS-sensitive damage hotspots at discrete locations. This is not completely unexpected, since these two chemicals induce RS with distinct mechanisms. APH inhibits DNA polymerases α and slows DNA polymerization during replication, generating stretches of single stand DNA at stalled forks [14, 16, 49]. Thus, APH is expected to cause forks to stall or collapse at vulnerable regions containing natural barriers for DNA polymerases. These regions likely require additional efforts to avoid the pausing or dissociation of polymerases. Consistently, several studies have shown that specialized DNA polymerases, including Pol η, Pol **ζ**, and Pol κ that facilitate DNA synthesis and promote the stability of APH-inducible FSs [50–53]. In contrast, RS induced by the DNA methylating agent MMS is more complex. Although MMS is capable of reacting with a number of nucleophilic sites on DNA including ring nitrogen and exocyclic oxygen on purines and pyrimidines, the reactivity towards electrophiles varies substantially by the position of the nucleotide, whether the nucleotide is at the major or minor groove, and whether the DNA is single or double stranded [54]. Consequently, it is difficult to pinpoint where the methylation adducts are formed. HU reduces or depletes the overall cellular nucleotide pool, and therefore is expected to stall all DNA synthesis and impact replication more globally. In agreement with this view, we find that HU induces several times more damage sites than other treatments. HU induces γH2AX binding hotspots at regions overlapping with APH or MMS treated samples, but this overlap only accounts for a small portion of data set due to the large peak numbers.

We observed that SINEs are enriched in γH2AX binding sites induced by all three treatments (Fig. 3), suggesting that SINEs may contain features that easily stall DNA polymerases. One such feature may be the poly (dA:dT) tracts at the 3′ end of SINEs, which have been implicated as a natural replication barriers and is a common cause for DNA breakage in murine lymphocytes [21]. Cumulating evidence indicates that SINEs regulate gene expression, affect chromatin structure, and are involved in genome rearrangement [55, 56], and therefore they have been implicated in many diseases including cancer [57]. It will be interesting to investigate the potential role of RS-induced SINE instability in disease development.

Despite different localizations of γH2AX binding, we find that they share a few obvious common features. First, all three conditions induce γH2AX binding at regions with the median transcript length longer than the median human transcript size (Fig. 2), indicating that regions with large transcripts are prone to break under RS. It has been shown that transcription of large genes often requires more than one complete cell cycle to complete. Collisions of transcription machinery with a replication fork and the formation of R-loops impede fork movement, causing FS instability [10]. Thus, our results reinforce transcription/replication collision as a crucial theme causing RS regardless of the RS mechanism.

In addition to increased binding at long genes, we also find that APH, HU, and MMS-induced γH2AX binding shows depletion of H3K9Ac and H3K4me3 marks, while being slightly enriched with H3K27me3 (Fig. 4), suggesting that chromatin within FSs may be more compact than non-fragile regions. It has been postulated that epigenetic feature regulates replication density and timing, with compact chromatic regions being poorly represented at replication initiation regions [12, 39]. In support of this, previous report shows that the six most break-prone human CFSs display an epigenetic pattern of histone hypoacetylation [11]. The same study also examines H3K9Ac acetylation pattern of large genes and find that acetylation coverage of large genes is substantially lower than that of the human genome on average. Our results therefore extend this finding to genome-wide FSs and support that compact chromatin may be a common epigenetic feature contributing to FS instability.

Previous research suggests that unprogrammed formation of R-loops impairs fork progression, causing fork stalling that contributes to DSB formation [58, 59]. A recent study has reported widespread R-loop formation at unmethylated CGI promoters in the human genome [60]. Therefore, our observation that γH2AX peaks flank but are not located at CGIs and TSSs is somewhat surprising (Fig. 5). In order to explain this observation, it is worth revisiting studies of mapping γH2AX distribution after DSB induction. DSBs trigger H2AX phosphorylation over large domains (0.5 to 2 Mb) surrounding the DSB [48]. Anti-correlation between RNA Pol II occupancy and γH2AX enrichment has been observed in both *S. cerevisiae* and the human U2OS cell line [48, 61], suggesting that TSSs and promoter regions may be particularly resistant to either the establishment or maintenance of H2AX phosphorylation. In addition, γH2AX enrichment at transcriptionally repressed genes seems to be dependent on HDACs [61]. Thus, it is highly likely that specialized chromatin structures at TSSs and CGIs prevent γH2AX accumulation despite R-loop formation. It will be interesting to determine the role of γH2AX depletion and specialized chromatin in stabilizing stalled forks at TSSs.

In conclusion, our study demonstrates that different types of replication stresses produce γH2AX binding at non-overlapping loci. By characterizing sequence and epigenetic features of these loci, our analysis provides a global view of the characteristics of genomic regions sensitive to various replication stress conditions. It is conceivable that cells may use different molecular mechanisms involving different protein molecules and repair pathways to rescue forks stalled at different types of fragile sequences. Since chromosome rearrangements found in cancer cells often result from genome instability caused by RS, deciphering the molecular mechanisms protecting RS-induced genome stability represents an important issue in the field.

## Methods

### Cell culture

Human B-lymphocyte cell line (GM07027) was obtained from Coriell Institute. 174xCEM was obtained from American Type Culture Collection (ATCC). GM07027 and 174xCEM lymphocyte cells were cultured in suspension and passaged in the RPMI1640 medium (Life Technologies) supplemented with 2 mM L-glutamine and 15% fetal bovine serum (Atlanta Biologicals) at 37 °C under 5% CO_2_. HeLa and HEK293T cells (ATCC) were cultured in DMEM media supplemented with 10% cosmic calf serum (ThermoFisher) at 37 °C containing 5% CO2. No antibiotics were used to avoid possible antibiotics-induced stress.

### Antibodies and drugs

The following antibodies were used: γH2AX (Active Motif, 39117), H3 (Active Motif, 31277), CHK1 (Santa Cruz, sc-8408), CHK1pS317 (Cell Signaling Technology, 2344P), Actin (Sigma, A5441), BrdU (BD Biosciences, 347,580). Secondary antibodies were as follows: horseradish peroxidase-conjugated anti-mouse IgG (BD Biosciences, 554002) and anti-rabbit IgG (Vector Laboratories, PI-1000) for western blotting. Drugs used in this study include: HU (ThermoFisher Scientific), APH (Sigma), MMS (ThermoFisher Scientific), and VE-821 (Selleckchem).

### Flow cytometry

All flow cytometry analysis was performed with a Beckman Coulter Gallios flow cytometer. Samples were filtered through 40 μm nylon mesh prior to flow cytometry. All analysis was performed using Kaluza analysis software (Beckman Coulter).

#### BrdU incorporation

Cells were treated with 2 mM HU, 0.3 μM APH or 200 μM MMS for 24 h, and then incubated with 10 μM BrdU at 37 °C under 5% CO_2_ for 30 min. Cells were collected by centrifugation, washed with cold PBS once, resuspended and fixed with 70% ethanol at 4 °C overnight. Cells were then washed with PBS and resuspended in 2 N HCl supplemented with 0.5% Triton X-100 and incubated for 30 min at room temperature. Cells were centrifuged to remove HCl and then neutralized with 0.1 M sodium borate buffer pH 8.5. Cells were washed by cold PBS, resuspended in BrdU staining buffer (PBS, 0.5% Tween-20, 1% BSA), incubated with anti-BrdU antibody for 1 h at room temperature, followed by cold PBS wash and incubation with the secondary antibody goat anti-mouse Alexa Fluor 488 (ThermoFisher Scientific, A11029) for 1 h at room temperature. After centrifugation, cell pellets were washed by PBS and stained with propidium iodide (PI) solution (PBS, 0.1% NP-40, 2% fetal bovine serum, 50 μg/ml PI and 50 μg/ml RNase A) for 30 min in dark.

#### Cell cycle analysis

Cell cycle was analyzed by PI staining. Briefly, cells were treated with genotoxic drugs for 24 h, and then collected by centrifugation, washed with cold PBS once, resuspended and fixed with 70% ethanol at 4 °C for 1 h. Cells were then washed by PBS once, resuspended with PI solution and incubated at 37 °C for 1 h.

#### Apoptosis assay

Quantification of apoptosis was measured by annexin V staining (BioLegend, #640905) by following as the manufacturer’s instruction. Briefly, after cells were treated with 2 mM HU, 0.3 μM APH or 200 μM MMS for 24 h, cells were collected by centrifugation, and washed with cold PBS, resuspended in 100 μl binding buffer (10 mM HEPES pH 7.4, 140 mM NaCl and 2.5 mM CaCl_2_) supplemented with 5 μl annexin V-FITC solution, and incubated for 20 min at room temperature in the dark. Binding buffer (BioLegend, 400 μl) was then added to each sample and subject to FACS.

### ChIP-seq sample preparation

Cells were treated with 2 mM HU, 0.3 μM APH or 200 μM MMS for 24 h, collected by centrifugation, resuspended in PBS and crosslinked with 1% formaldehyde for 15 min at r.t.. Crosslinking was stopped by 0.2 M glycine, cells were centrifuged, resuspended in lysis buffer (50 mM Tris-HCl pH 8.0, 1% Triton X-100, 1% SDS, protease inhibitor cocktail containing 1 mM AEBSF, 0.3 μM aprotinin, 50 μM bestatin, 10 μM E-64, 10 μM leupeptin, 5 μM pepstain and 1 mM PMSF), sonicated on ice for 10 times in 10 s pulses to obtain DNA fragments 100–500 bp in length, and centrifuged again at 4 °C for 10 min at 20,000 g. The supernatant was then diluted with four volumes of dilution buffer (0.01% SDS, 1.1% Triton X-100, 1.2 mM EDTA, 16.7 mM Tris-HCl pH 8.0, 150 mM NaCl, protease inhibitors) and precleared with protein A beads (Roche) at 4 °C for 1 h. Precleared lysates were incubated with anti-γH2AX (Active Motif, #39117) at 4 °C for overnight, followed by the addition of Protein A beads. After additional 3 h incubation at 4 °C, beads were washed sequentially with 1 ml of buffer A (0.1% SDS, 1% Triton X-100, 2 mM EDTA, 20 mM Tris-HCl pH 8.0, 150 mM NaCl), buffer B (0.1% SDS, 1% Triton X-100, 2 mM EDTA, 20 mM Tris-HCl pH 8.0, 500 mM NaCl), buffer C (250 mM LiCl, 1% NP-40, 1% Na-deoxycholate, 1 mM EDTA, 10 mM Tris-HCl pH 8.0), and buffer D (1 mM EDTA, 10 mM Tris-HCl pH 8.0) for 5 min at 4 °C with rotation. Beads were then washed with buffer D again for 5 min, and eluted with 300 μl elution buffer (1% SDS, 100 mM NaHCO_3_) at 50 °C for 15 min. Elutes were reverse crosslinked in 200 mM NaCl and 20 μg Protease K at 65 °C overnight. DNA was then precipitated by ethanol and precipitated DNA was used for ChIP-seq library construction.

### NGS library preparation and sequencing

Libraries were prepared according to Illumina’s TruSeq® ChIP Sample Preparation Guide (Part# 15023092 Rev. B). Briefly, ChIP DNA was end-repaired using a combination of T4 DNA polymerase, *E. coli* DNA Pol I large fragment (Klenow polymerase) and T4 polynucleotide kinase. The blunt, phosphorylated ends were treated with Klenow fragment (32 to 52 exo minus) and dATP to yield a protruding 3- ‘A’ base for ligation of Illumina’s adapters which have a single ‘T’ base overhang at the 3′ end. After adapter ligation, DNA fragments with sizes of 250–300 bp were selected on 2% agarose gels and were PCR amplified with Illumina primers for 18 cycles. The libraries were captured on an Illumina flow cell for cluster generation and sequenced on HiSeq 2500 (Illumina) with paired-end 100 bp read length following the manufacturer’s protocols. For each genotoxin, two independent treatments were performed, followed by independent ChIP experiments. This resulted in a total of eight ChIP samples (untreated, APH, HU, MMS) that were sequenced simultaneously.

### ChIP-seq reads processing and sequence analysis

Prior to sequence analysis, adaptor sequences in reads were trimmed. Paired-end reads in fastq format were aligned to the GRCh38 reference genome using Bowtie2 default settings [62]. Reads were checked for quality control using Samtools [63], and reads below q40 were removed. PCR duplicates were also removed. Following alignment, broad peaks were called using MACS2 peak-calling program [31] (with settings --broad --no-model, −-broad-cutoff 10e-3 -p) to give the final peak list per replicate. Shift size was determined using gel quantification from library quality controls. Shift sizes were determined to be: APH-treated replicate 1: 251; APH-treated replicate 2: 257; HU-treated replicate 1: 248; HU-treated replicate 2: 243; MMS-treated replicate 1: 222; MMS-treated replicate 2: 241; Untreated replicate 1: 214; Untreated replicate 2: 229. Blacklisted regions were removed from analysis [64]. Reproducibility between replicates was assessed using Spearman Rank Correlation of tags per 1000 bp bin. All ChIP-seq data is available at the Gene Express Omnibus at accession no. GSE113020.

Enrichment or depletion of γH2AX ChIP-seq peaks in repetitive elements, CGIs, and CFSs were assessed using 1000 iteration permutation analysis with the regioneR Bioconductor package [65]. Repetitive elements were defined by RepeatMasker [37], which uses RepBase Update, the database of repetitive sequences throughout multiple species to define repetitive sequences [66]. This database contains transposable elements (SINES, LINES, DNA-transposons, and LTRs), and non-mobile DNA repeat elements which include the canonical TTAGGG telomere sequence (simple repeats/microsatellites), regions of low complexity such as the known fragile poly-T motif, and (x) RNA sources found throughout the genome. Positions and categories of repetitive elements were obtained from the RepeatMasker data set [37]. Positions of CGIs were obtained from the CGI track in the UCSC Genome Browser [42]. CFSs in human lymphocytes [15, 18, 67] were sorted using the G-band positions from the UCSC Chromosome band track [68, 69]. The NCBI RefSeq dataset was used for gene lengths, TSS, and TTS analyses [70]. Gene length was analyzed using a Kruskal-Wallis test and post-hoc paired Wilcoxon signed-rank test with a Holm-Bonferroni correction for family-wise error. Graphs for gene length were generated using the ggplot2 R package [71]. Graphs for ChIP-seq data relationships to TSS and histone marks were generated using Deeptools2 [72]. Histone mark data was taken from GSM733677 (H3K9ac), GSM733708 (H3K4me3), GSM945196 (H3K27me3), GSM733664 (H3K9me3) [64, 73]. Sample data was realigned to hg19 using identical Bowtie2 settings prior to comparison with histone marks.

## Additional file

Additional file 1: **Figures S1-S8.** (PPTX 986 kb)

Additional file 2: **Table S1.** List of Human CFSs used in this study. (XLSX 54 kb)

## Data Availability

All ChIP-seq data are deposited in GEO under accession number GSE113020.

## Abbreviations

APH: Aphidicolin
ATR: Ataxia Telangiectasia mutated and Rad-3 related
BrdU: 5-Bromo-2′-deoxyuridine
CFS: Common fragile site
CGI: GpG island
ChIP-seq: Chromatin immunoprecipitation sequencing
DSB: Double strand break
ERFS: Early replication fragile site
FS: Fragile site
HU: Hydroxyurea
LINE: Long interspersed nuclear elements
LTR: Long terminal repeats
MMS: Methyl methanesulfonate
PI: Propidium iodide
RS: Replication stress
SINE: Short interspersed nuclear elements
TSS: Transcription start site
TTS: Transcription termination site
