# Supplementary material for: Genome-wide mapping and profiling of γH2AX binding hotspots in response to different replication stress inducers
Source: BMC Genomics. 2019 Jul 12;20:579. doi: 10.1186/s12864-019-5934-4 (PMC6625122; doi:10.1186/s12864-019-5934-4)
Supplement: Supplementary file 1 — Figures S1-S8. (PPTX 986 kb) [file 12864_2019_5934_MOESM1_ESM.pptx]

## Slide 1
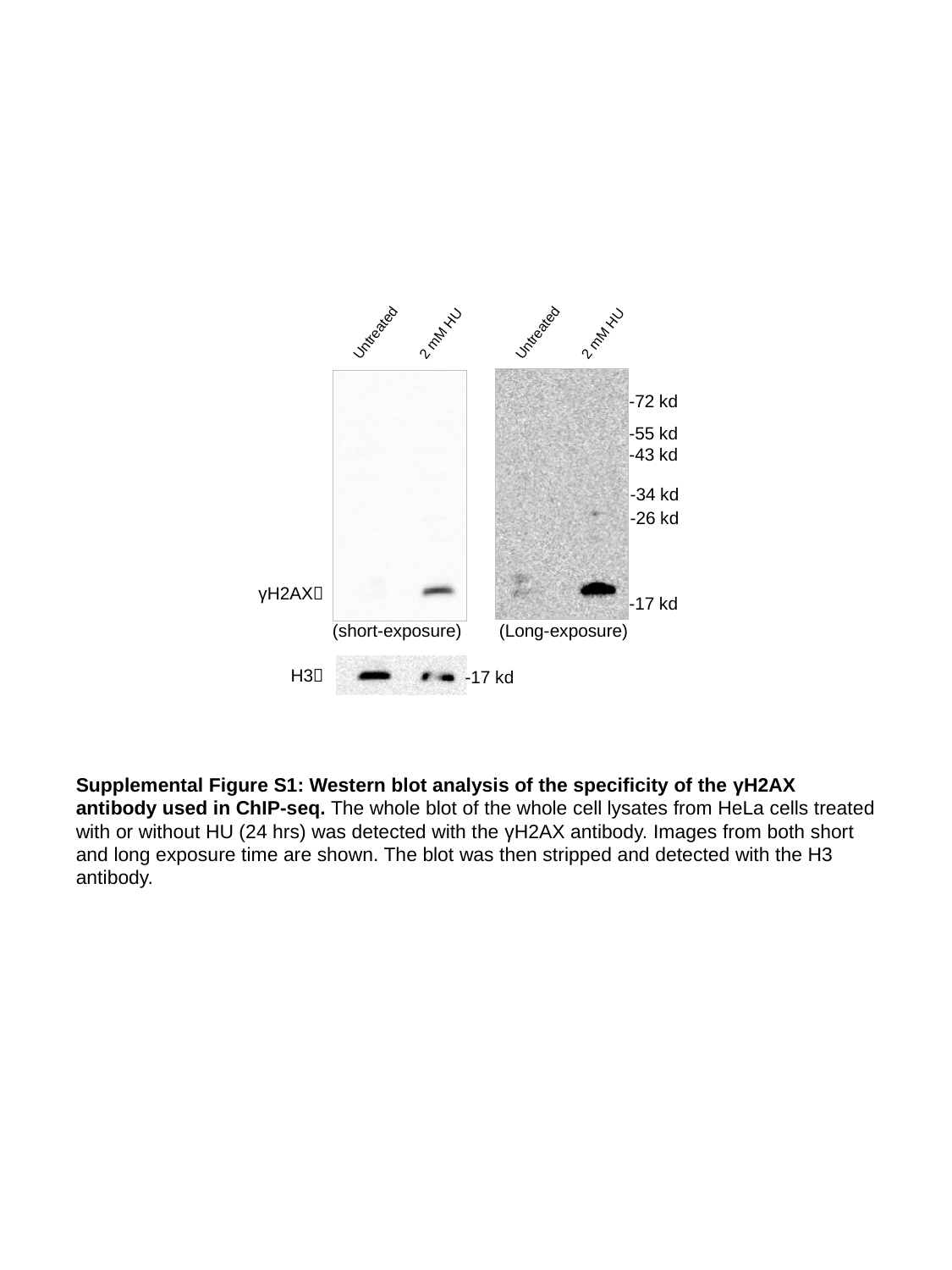

Untreated
2 mM HU
Untreated
2 mM HU
-72 kd
-55 kd
-43 kd
-34 kd
-26 kd
γH2AX
-17 kd
(short-exposure)
(Long-exposure)
H3
-17 kd
Supplemental Figure S1: Western blot analysis of the specificity of the γH2AX antibody used in ChIP-seq. The whole blot of the whole cell lysates from HeLa cells treated with or without HU (24 hrs) was detected with the γH2AX antibody. Images from both short and long exposure time are shown. The blot was then stripped and detected with the H3 antibody.

## Slide 2
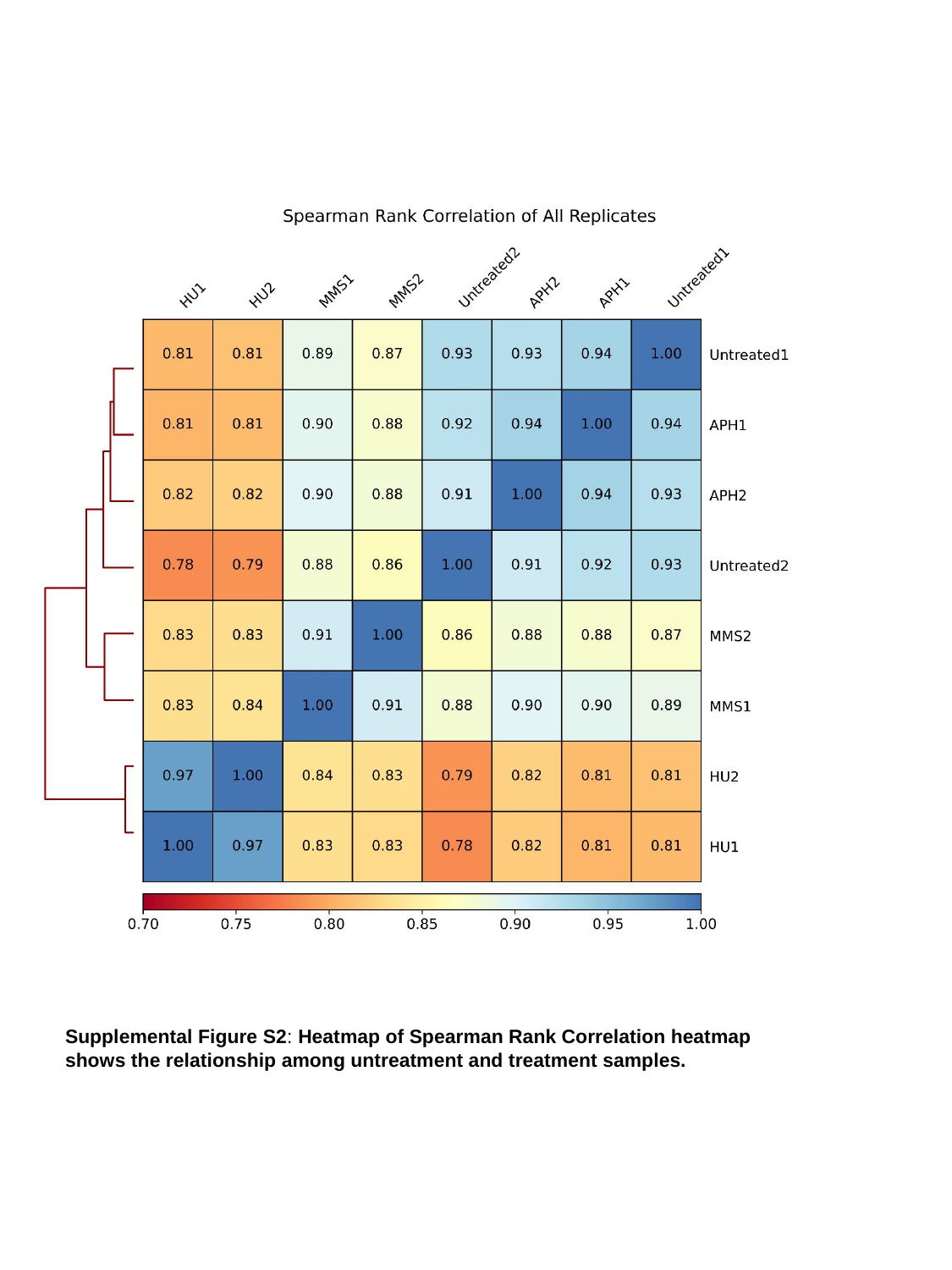

Supplemental Figure S2: Heatmap of Spearman Rank Correlation heatmap shows the relationship among untreatment and treatment samples.

## Slide 3
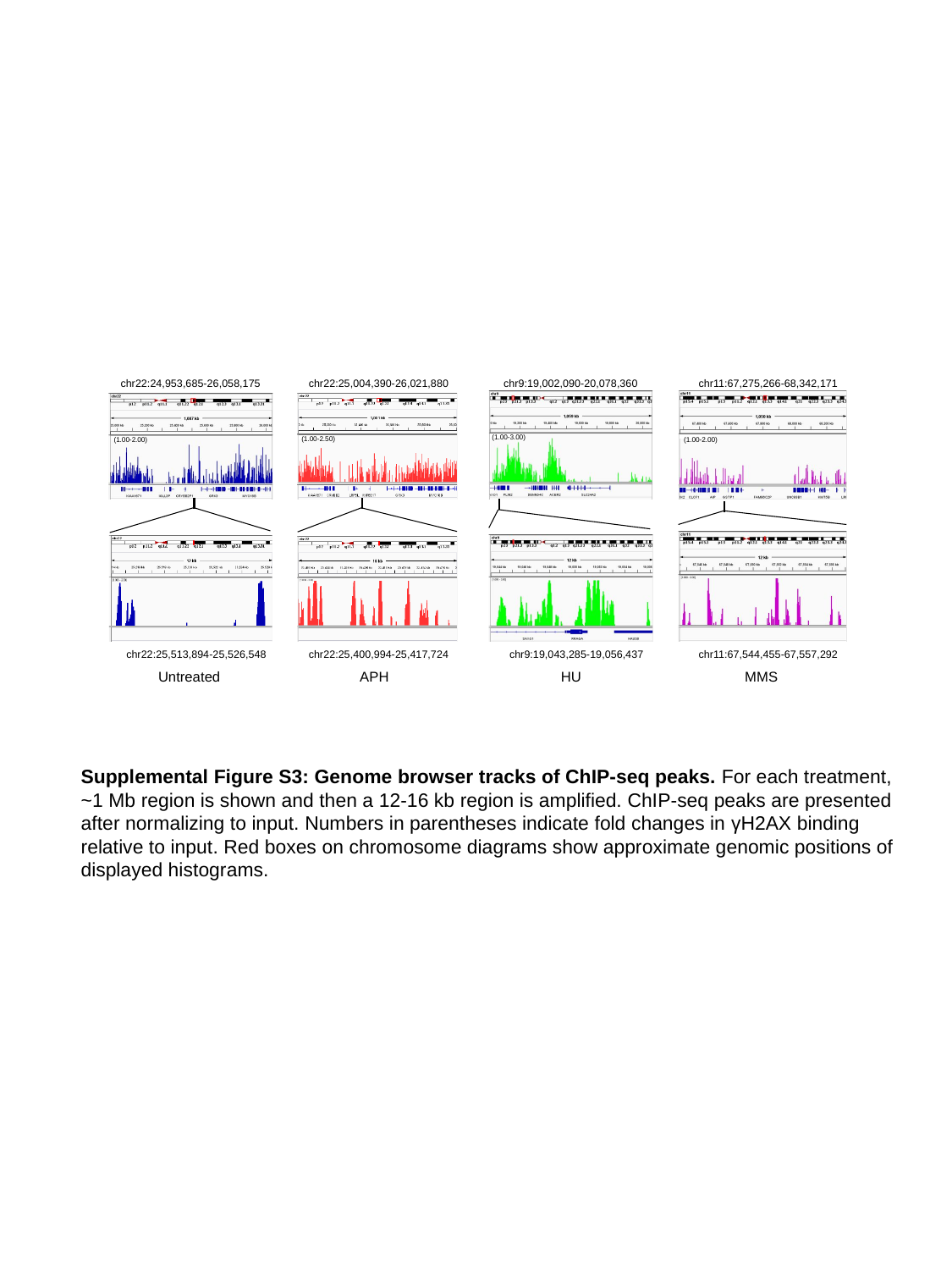

chr22:24,953,685-26,058,175
chr22:25,004,390-26,021,880
chr9:19,002,090-20,078,360
chr11:67,275,266-68,342,171
(1.00-3.00)
(1.00-2.50)
(1.00-2.00)
(1.00-2.00)
chr22:25,513,894-25,526,548
chr22:25,400,994-25,417,724
chr9:19,043,285-19,056,437
chr11:67,544,455-67,557,292
Untreated
APH
HU
MMS
Supplemental Figure S3: Genome browser tracks of ChIP-seq peaks. For each treatment, ~1 Mb region is shown and then a 12-16 kb region is amplified. ChIP-seq peaks are presented after normalizing to input. Numbers in parentheses indicate fold changes in γH2AX binding relative to input. Red boxes on chromosome diagrams show approximate genomic positions of displayed histograms.

## Slide 4
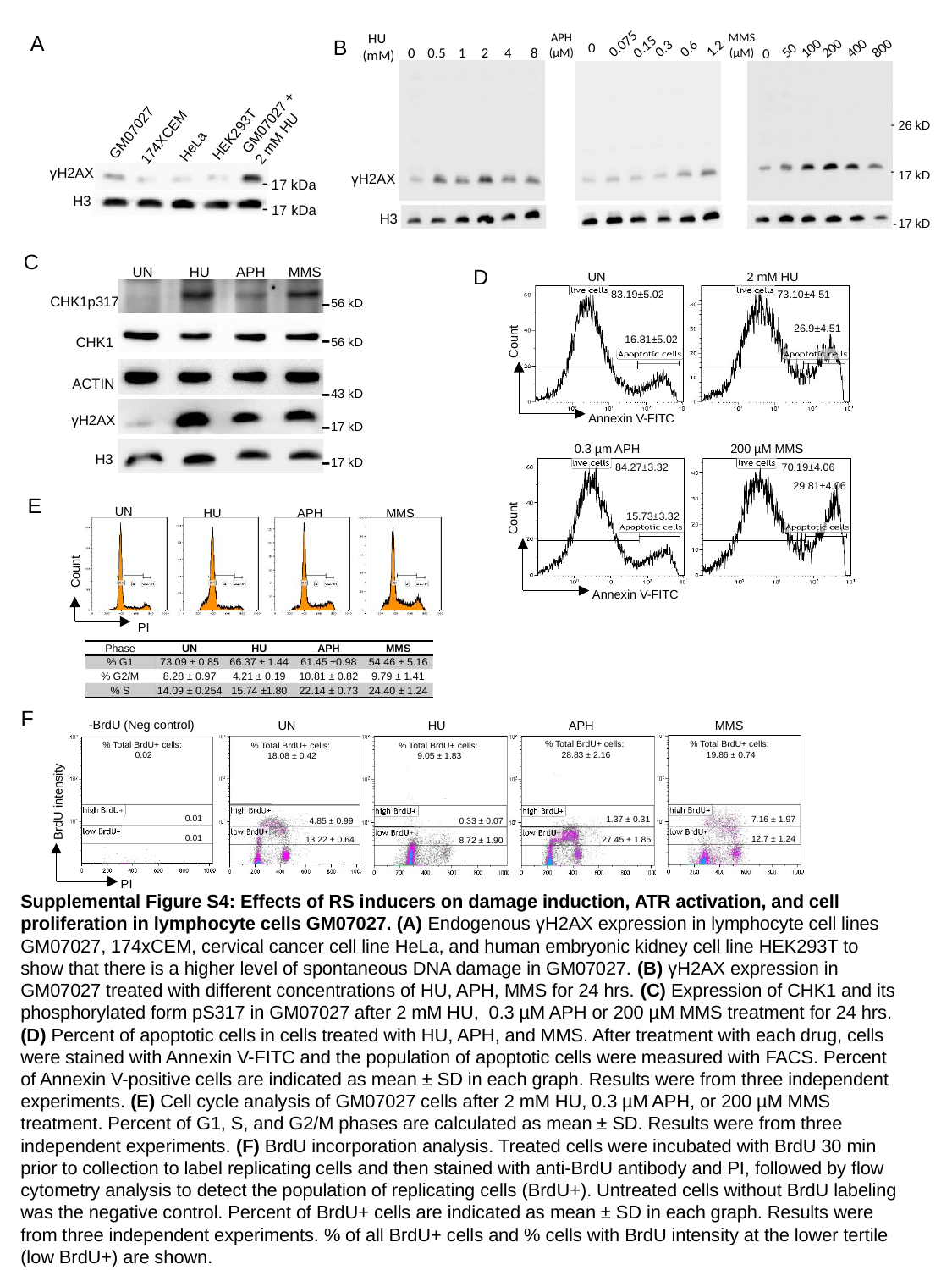

A
HU
(mM)
APH
(μM)
MMS
(μM)
26 kD
17 kD
17 kD
0 0.5 1 2 4 8
γH2AX
H3
-
-
-
0.075
0.15
0
100
200
400
800
0.3
0.6
1.2
50
0
B
GM07027 + 2 mM HU
GM07027
HEK293T
HeLa
174XCEM
γH2AX
17 kDa
H3
17 kDa
-
-
C
UN
HU
APH
MMS
CHK1p317
56 kD
CHK1
56 kD
ACTIN
43 kD
γH2AX
17 kD
H3
17 kD
-
-
-
-
-
D
UN
2 mM HU
83.19±5.02
73.10±4.51
Count
26.9±4.51
16.81±5.02
Annexin V-FITC
0.3 µm APH
200 µM MMS
84.27±3.32
70.19±4.06
29.81±4.06
15.73±3.32
Count
Annexin V-FITC
E
UN
HU
APH
MMS
Count
PI
| Phase | UN | HU | APH | MMS |
| --- | --- | --- | --- | --- |
| % G1 | 73.09 ± 0.85 | 66.37 ± 1.44 | 61.45 ±0.98 | 54.46 ± 5.16 |
| % G2/M | 8.28 ± 0.97 | 4.21 ± 0.19 | 10.81 ± 0.82 | 9.79 ± 1.41 |
| % S | 14.09 ± 0.254 | 15.74 ±1.80 | 22.14 ± 0.73 | 24.40 ± 1.24 |
F
-BrdU (Neg control)
UN
HU
APH
MMS
% Total BrdU+ cells: 19.86 ± 0.74
% Total BrdU+ cells: 28.83 ± 2.16
% Total BrdU+ cells: 0.02
% Total BrdU+ cells: 18.08 ± 0.42
% Total BrdU+ cells: 9.05 ± 1.83
BrdU intensity
0.01
1.37 ± 0.31
7.16 ± 1.97
4.85 ± 0.99
0.33 ± 0.07
0.01
12.7 ± 1.24
13.22 ± 0.64
27.45 ± 1.85
8.72 ± 1.90
PI
Supplemental Figure S4: Effects of RS inducers on damage induction, ATR activation, and cell proliferation in lymphocyte cells GM07027. (A) Endogenous γH2AX expression in lymphocyte cell lines GM07027, 174xCEM, cervical cancer cell line HeLa, and human embryonic kidney cell line HEK293T to show that there is a higher level of spontaneous DNA damage in GM07027. (B) γH2AX expression in GM07027 treated with different concentrations of HU, APH, MMS for 24 hrs. (C) Expression of CHK1 and its phosphorylated form pS317 in GM07027 after 2 mM HU, 0.3 µM APH or 200 µM MMS treatment for 24 hrs. (D) Percent of apoptotic cells in cells treated with HU, APH, and MMS. After treatment with each drug, cells were stained with Annexin V-FITC and the population of apoptotic cells were measured with FACS. Percent of Annexin V-positive cells are indicated as mean ± SD in each graph. Results were from three independent experiments. (E) Cell cycle analysis of GM07027 cells after 2 mM HU, 0.3 µM APH, or 200 µM MMS treatment. Percent of G1, S, and G2/M phases are calculated as mean ± SD. Results were from three independent experiments. (F) BrdU incorporation analysis. Treated cells were incubated with BrdU 30 min prior to collection to label replicating cells and then stained with anti-BrdU antibody and PI, followed by flow cytometry analysis to detect the population of replicating cells (BrdU+). Untreated cells without BrdU labeling was the negative control. Percent of BrdU+ cells are indicated as mean ± SD in each graph. Results were from three independent experiments. % of all BrdU+ cells and % cells with BrdU intensity at the lower tertile (low BrdU+) are shown.

## Slide 5
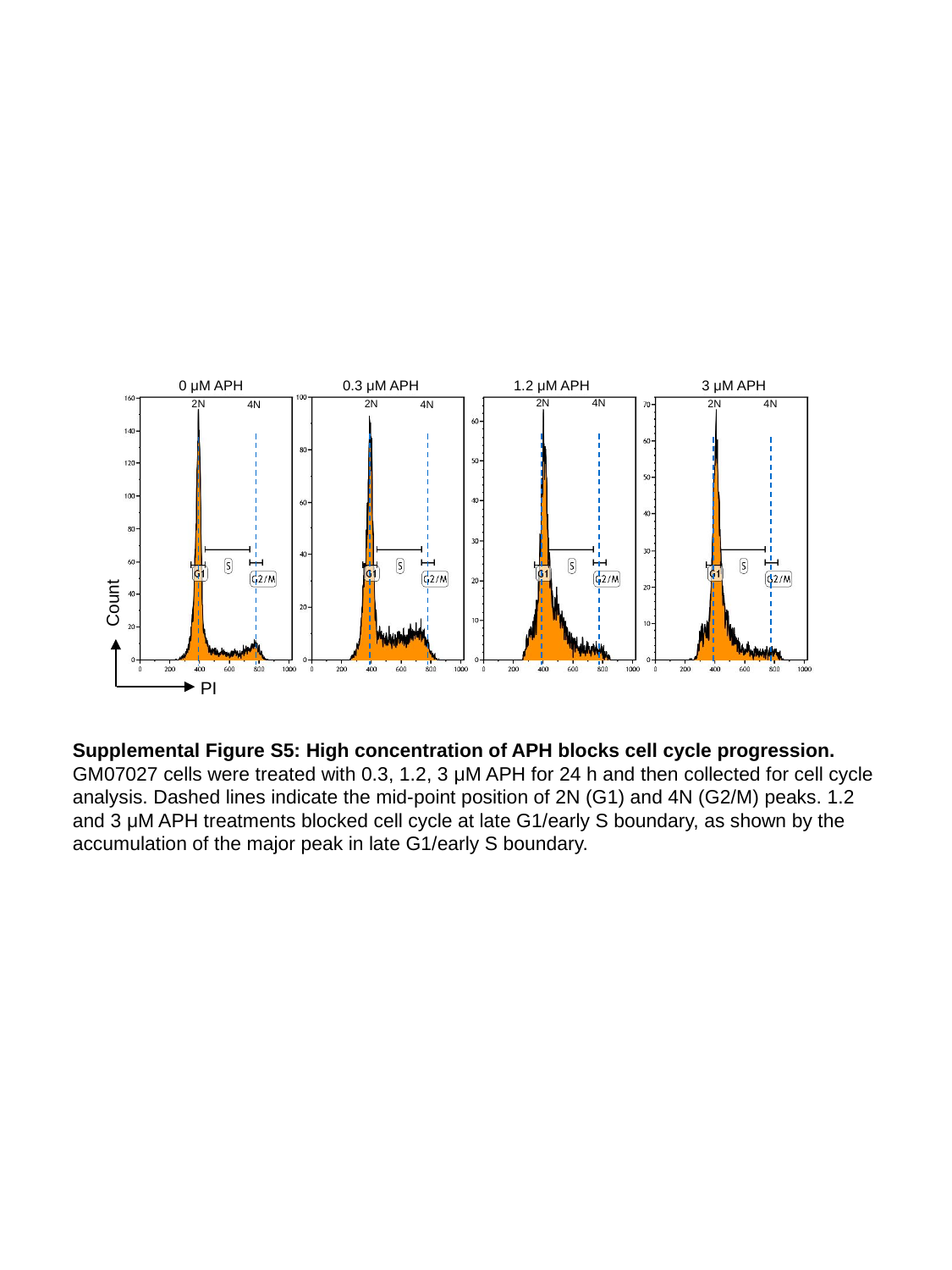

0 μM APH
0.3 μM APH
1.2 μM APH
3 μM APH
Count
PI
2N
4N
2N
2N
4N
2N
4N
4N
Supplemental Figure S5: High concentration of APH blocks cell cycle progression. GM07027 cells were treated with 0.3, 1.2, 3 μM APH for 24 h and then collected for cell cycle analysis. Dashed lines indicate the mid-point position of 2N (G1) and 4N (G2/M) peaks. 1.2 and 3 μM APH treatments blocked cell cycle at late G1/early S boundary, as shown by the accumulation of the major peak in late G1/early S boundary.

## Slide 6
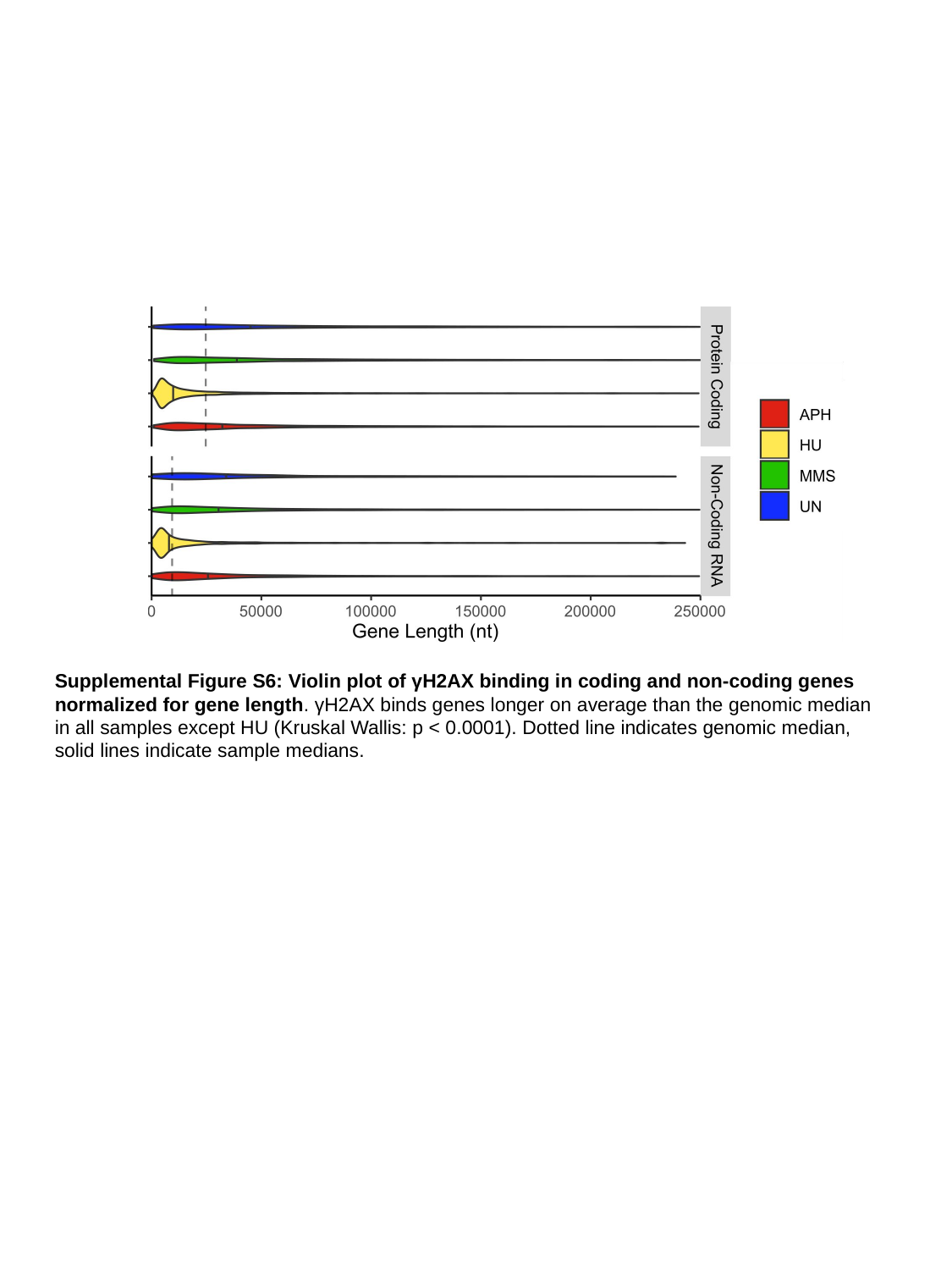

Supplemental Figure S6: Violin plot of γH2AX binding in coding and non-coding genes normalized for gene length. γH2AX binds genes longer on average than the genomic median in all samples except HU (Kruskal Wallis: p < 0.0001). Dotted line indicates genomic median, solid lines indicate sample medians.

## Slide 7
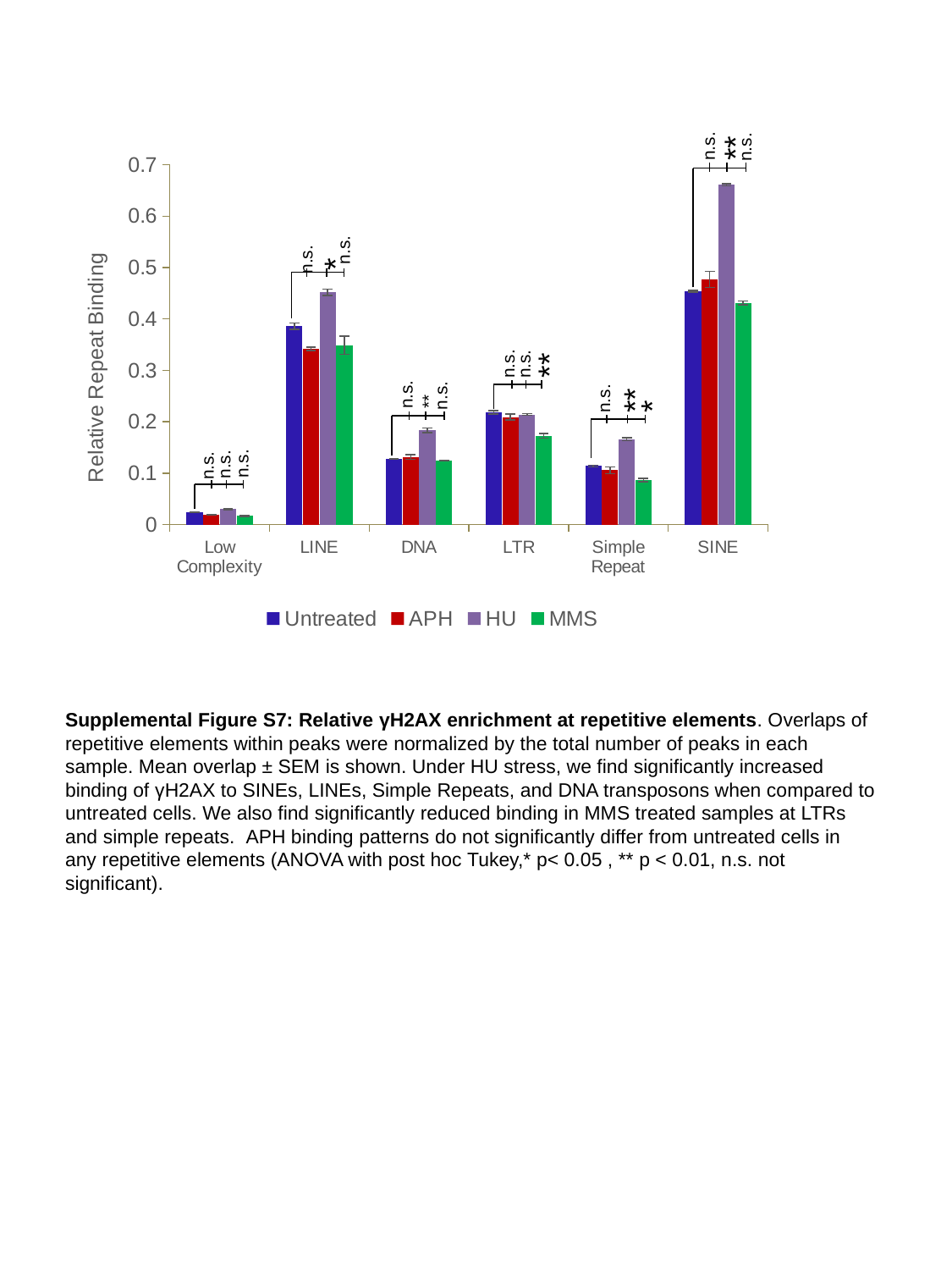

**
n.s.
n.s.
### Chart
| Category | Untreated | APH | HU | MMS |
|---|---|---|---|---|
| Low Complexity | 0.024085972452561805 | 0.018866424686104277 | 0.030025214306321303 | 0.016923260787143395 |
| LINE | 0.385764526537736 | 0.3419233275279881 | 0.4515876578155229 | 0.3488186142943418 |
| DNA | 0.12714284212574145 | 0.1314090094195266 | 0.1833809184314943 | 0.12489116572930575 |
| LTR | 0.21820503748529224 | 0.2091666348885158 | 0.21422200878063902 | 0.17258661409652945 |
| Simple Repeat | 0.11388176210177411 | 0.10558537405377538 | 0.16606887800523895 | 0.08677257121620144 |
| SINE | 0.4539726192175044 | 0.47703258282190625 | 0.6607725071964368 | 0.43129110788417185 |n.s.
*
n.s.
*
**
n.s.
**
n.s.
n.s.
n.s.
n.s.
**
n.s.
n.s.
n.s.
Supplemental Figure S7: Relative γH2AX enrichment at repetitive elements. Overlaps of repetitive elements within peaks were normalized by the total number of peaks in each sample. Mean overlap ± SEM is shown. Under HU stress, we find significantly increased binding of γH2AX to SINEs, LINEs, Simple Repeats, and DNA transposons when compared to untreated cells. We also find significantly reduced binding in MMS treated samples at LTRs and simple repeats.  APH binding patterns do not significantly differ from untreated cells in any repetitive elements (ANOVA with post hoc Tukey,* p< 0.05 , ** p < 0.01, n.s. not significant).

## Slide 8
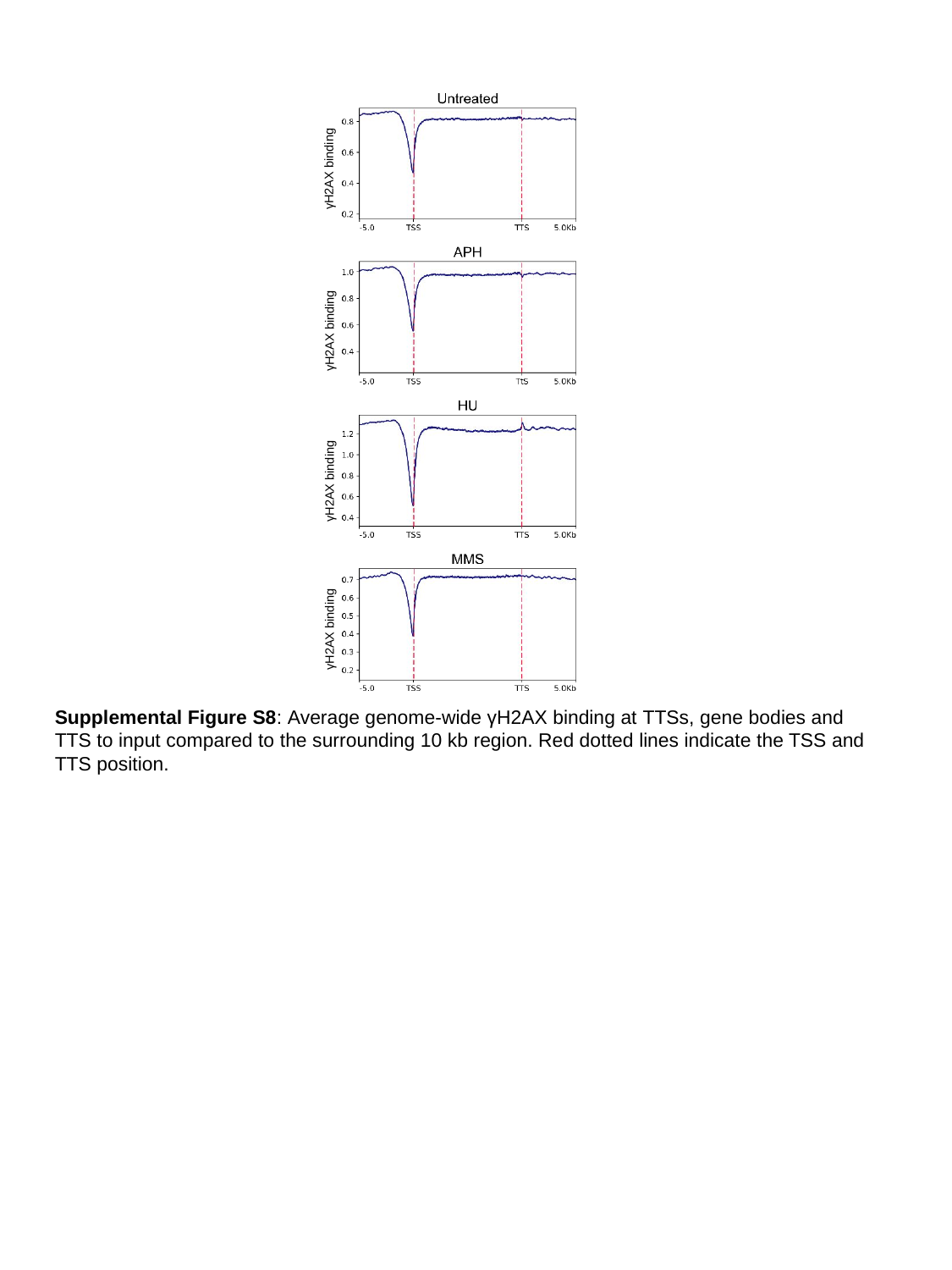

γH2AX binding
γH2AX binding
γH2AX binding
γH2AX binding
Supplemental Figure S8: Average genome-wide γH2AX binding at TTSs, gene bodies and TTS to input compared to the surrounding 10 kb region. Red dotted lines indicate the TSS and TTS position.
